# C-Reactive Protein-to-Albumin Ratio Predicts Sepsis and Prognosis in Patients with Severe Burn Injury

**DOI:** 10.1155/2021/6621101

**Published:** 2021-03-24

**Authors:** Yaohua Yu, Weiwei Wu, Yanyan Dong, Jiliang Li

**Affiliations:** ^1^Department of Plastic and Burn Surgery, Hwa Mei Hospital, University of Chinese Academy of Sciences, No. 41 Xibei Road, Haishu District, Ningbo City, 315000 Zhejiang Province, China; ^2^Ningbo Institute of Life and Health Industry, University of Chinese Academy of Sciences, China; ^3^Ningbo College of Health Sciences, China

## Abstract

**Background:**

Sepsis is a leading cause of mortality among severe burns. This study was conducted to investigate the predictive role of C-reactive protein-to-albumin ratio (CAR) for sepsis and prognosis in severe burns.

**Methods:**

Patients with severe burn injuries from 2013 to 2017 were enrolled and divided into septic and nonseptic groups based on the presence of sepsis within 30 days postburn. Independent risk factors for sepsis were performed by the univariate and multivariate logistic regression analyses. The association between CAR level at admission and postburn 30-day mortality was designed via the Kaplan–Meier method.

**Results:**

Of all the 196 enrolled patients, 83 patients developed sepsis within 30 days postburn injury, with an incidence of 42.3%. TBSA percentage (OR: 1.65, 95% CI: 1.17-2.32, *P* = 0.014) and CAR at admission (OR: 2.25, 95% CI: 1.33-3.56, *P* = 0.009) were the two independent risk factors for sepsis in severe burns by the multivariate logistic regression analysis. A higher CAR level (≥1.66) at admission was associated with a lower postburn 30-day survival rate (*P* = 0.005).

**Conclusions:**

The CAR level at admission was an independent risk factor for sepsis and prognosis in severe burns.

## 1. Introduction

Globally, burn injury is one of the important causes of morbidity and mortality [1]. A severe burn trauma is a very common acute injury with the characteristics of aggressiveness [2]. Despite an advancing understanding of postburn resuscitation, organ support therapy, and wound treatment, patients with severe burn injuries are commonly accompanied with sepsis, septic shock, and organ dysfunction [3]. As reported in previous studies, sepsis is a frequent complication in patients with severe burn injury, which is closely associated with an increased incidence of morbidity and mortality [4]. Sepsis is a leading cause of mortality among severe burns, particularly when complicated by septic shock or multiple organ dysfunction syndromes (MODS). Due to the advancing understanding of pathophysiology and therapeutic strategies, the mortality rate has decreased significantly over the decades. However, the reported mortality in burn injury patients with sepsis is still as high as 20.3% [5]. As a result, early prediction for sepsis in severe burns is critically important for prognosis improvement. However, the diagnosis of sepsis in burn injuries is sometimes difficult due to the obscure diagnostic criteria and classical signs.

C-reactive protein (CRP), an acute-phase protein, is closely associated with systemic inflammatory status [6]. A recent study by Gulhan et al. [7] indicates CRP (≥6 mg/dL) as a risk factor for developing sepsis in pediatric patients with burn injuries. However, some others hold the view that CRP is a confounding factor in identifying sepsis in burn patients because the chronic inflammatory response is part of the normal stress response in patients with burn injuries [8]. A previous study has shown that the albumin (Alb) level may be a sensitive and specific biomarker for severity and a prognostic factor for mortality in burn patients [9]. CRP-to-Alb ratio (CAR), based on CRP and Alb, presents not only the inflammatory but also the nutritional status. With improved consistency when comparing with CRP or Alb alone, CAR is widely reported to be a prognostic factor in a variety of studies, including lung cancer [10], ovarian cancer [11], esophageal cancer [12], and critically ill patients admitted to the intensive care unit (ICU) [13]. However, to our knowledge, no studies have illustrated the role of CAR in sepsis and prognosis prediction in severe burns. We herein firstly investigated the prognostic values of CAR in severe burns.

## 2. Material and Methods

### 2.1. Patients

**Figure 1:**
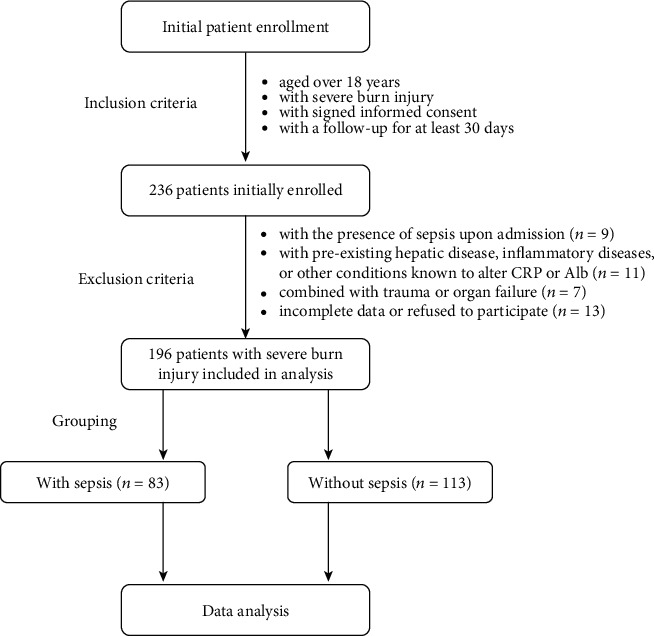
Flow chart. CRP: C-reactive protein; Alb: albumin.

This was a retrospective study with the approval of the Medical Institutional Ethics Committee of our hospital (approval date: 2013.03.05, No. PJ-KY-NBEY-2013-002-08). Patients with severe burn injuries at the Department of Plastic and Burn Surgery, Hwa Mei Hospital, University of Chinese Academy of Sciences, from 2013 to 2017 were initially enrolled. The inclusion criteria were described as follows: (1) aged over 18 years, (2) with severe burn injury, (3) with signed informed consent, and (4) with a follow-up for at least 30 days. Exclusion criteria were as follows: (1) with the presence of sepsis upon admission; (2) with preexisting hepatic disease (i.e., liver cirrhosis and hepatitis), inflammatory diseases (i.e., osteoarthritis and inflammatory bowel disease), or other conditions (i.e., acute pancreatitis, acute infection, myocardial infarction, malignancies, malnutrition, and nephrosis) known to alter CRP or Alb; (3) combined with trauma or organ failure; and (4) incomplete data or refused to participate. As shown in the flow chart in Figure 1, 236 patients with severe burn injuries were initially enrolled and 40 were excluded according to the exclusion criteria. A total of 196 patients were enrolled in the final data analysis.

### 2.2. Treatment and Definitions

The treatment and management of enrolled patients were carried out following the relevant guidelines (including fluid resuscitation, nutritional support, surgery, management of inhalation injury, and prevention and treatment of infection). All the enrolled patients receive burn surgery (eschar excision and allogeneic skin covering for the first time, followed by repeatedly autologous “stamp” or mesh skin graft) within 30 days postburn. The fluid resuscitation was performed according to the protocol by the Third Military Medical University (TMMU) formula [14]. In summary, 1 mL of lactated Ringer's solution and 0.5 mL of plasma per 1% TBSA burn area per kilogram (kg) were used for the first 24 h; 0.5 mL of lactated Ringer's solution and 0.25 mL of plasma per 1% TBSA burn area per kilogram (kg) were used for the next 24 h. In addition, 2 L water (as a 5% glucose solution) was additionally added as a daily basic requirement. The target of fluid resuscitation was set as hourly urine output ≥ 0.5 mL/kg/h. Moreover, the hemodynamics targets by pulse indicator continuous cardiac output (PICCO) were set as cardiac index (CI) > 2.5 L/min/m^2^, intrathoracic blood volume index (ITBVI) > 600 mL/m^2^, and lactic acid < 2 mmol/L [15].

Severe burn injury (including extremely severe burn) in this study was defined as a % total burn surface area (TBSA) ≥ 30% or third-degree TBSA ≥ 10%. Sepsis was defined as life-threatening organ dysfunction which was caused by a dysregulated host response to infection according to the recommendations by the Third International Consensus Definitions for Sepsis and Septic Shock (Sepsis-3) [16]. Clinical criteria for sepsis were documented (or suspected) infection and an acute increase of ≥2 Sequential Organ Failure Assessment (SOFA) points (a proxy for organ dysfunction) [16]. Sepsis within 30 days after burn injury was the primary observational endpoint, while mortality was set as the second endpoint.

### 2.3. Data Collection

The following data were collected: (1) demographics, including age, gender distribution, and body mass index (BMI); (2) preoperative comorbidities, including diabetes mellitus, hypertension, chronic coronary disease (CCD), and chronic obstructive pulmonary disease (COPD); (3) clinical data, including burn causes, admission time from burn, first excision time from burn, burn index, abbreviated burn severity index (ABSI) [17], Acute Physiology and Chronic Health Evaluation II (APACHE II) score and SOFA score [18] at admission, TBSA percentage, third-degree TBSA percentage, presence of inhalation injury, mechanical ventilation, and ICU admission; and (4) laboratory variables at admission, including hemoglobin (Hb), platelet (Plt), white blood cell (WBC), hematocrit (Hct), procalcitonin (PCT), blood urea nitrogen (BUN), creatinine, tumor necrosis factor-*α* (TNF-*α*), CRP, and Alb. The burn index was calculated with the following formula: third-degree TBSA percentage + 1/2 second-degree TBSA percentage [19].

### 2.4. Statistical Analysis

Data analysis was performed using SPSS 19.0 (SPSS, Inc., IA, USA) and GraphPad 8.0 (GraphPad Inc., CA, USA). Continuous variables with normal distribution are presented as mean ± standard error (S.E.M.) and analyzed using the Student *t*-test. Continuous variables without normal distribution are presented as median with range and analyzed using the Mann–Whitney *U* test. Categorical data are presented as a number with percentage (*n*, %) and compared using the chi-squared test or Fisher exact test. The predictive and cut-off values of variables for sepsis were established by receiving operating characteristic (ROC) curves. Risk factors associated with sepsis occurrence in severe burns were designed based on the univariate and multivariate binary logistic regression analyses using enter method. Multicollinearity test which includes variance inflation factor (VIF) was also conducted to evaluate the multicollinearity among factors. Only those factors with a *P* value < 0.05 in univariate logistic analysis were further included in the multivariate logistic regression model. The association between the CAR level at admission and postburn 30-day mortality was designed via the Kaplan–Meier method and log-rank test. A *P* value of <0.05 was considered statistically different.

## 3. Results

### 3.1. Patient Characteristics

A total of 196 patients with severe burn injuries were enrolled in this study. As shown in Figure 1, 83 patients developed sepsis within 30 days of postburn injury, with an incidence of 42.3% (83/196). The mean age of the cohort was 42.5 years and most patients were males (62.2%, 122/196), with the main causes of thermal (80.6%, 158/196). The majority of patients (68.9%, 135/196) were admitted to ICU due to different reasons. Of the 135 patients admitted to ICU, 74 (54.8%) were with shock and 81 (60.0%) were with sepsis (see Table 1). The demographics and baseline characteristics were compared between patients with or without sepsis, which is presented in Table 2. Age, gender, BMI, causes, admission time from burn, preoperative comorbidities, APACHE II, and SOFA score at admission did not appear significantly different between sepsis and nonsepsis individuals (*P* > 0.05). The first excision time from burn (*P* = 0.037), burn index (*P* = 0.003), ABSI (*P* = 0.001), TBSA percentage (*P* < 0.001), and third-degree TBSA percentage (*P* = 0.006) were significantly higher in septic patients when comparing with nonseptic patients. Patients with the presence of inhalation injury were more susceptible to sepsis than those without inhalation injury (*P* = 0.021). Patients with sepsis were associated with higher rates of mechanical ventilation (*P* = 0.005) and ICU admission (*P* < 0.001), which was easily understandable.  Table 3 demonstrates the comparative results of laboratory variables associated with sepsis in severe burns. Compared with nonsepsis patients, the serum expressions of TNF-*α* (*P* = 0.012), PCT (*P* < 0.001), and CAR (*P* < 0.001) at admission were significantly higher in sepsis patients. The levels of Hb, Plt, WBC, Hct, creatinine, and BUN did not differ significantly between these two groups (*P* > 0.05).

### 3.2. Predictors for Sepsis

**Figure 2:**
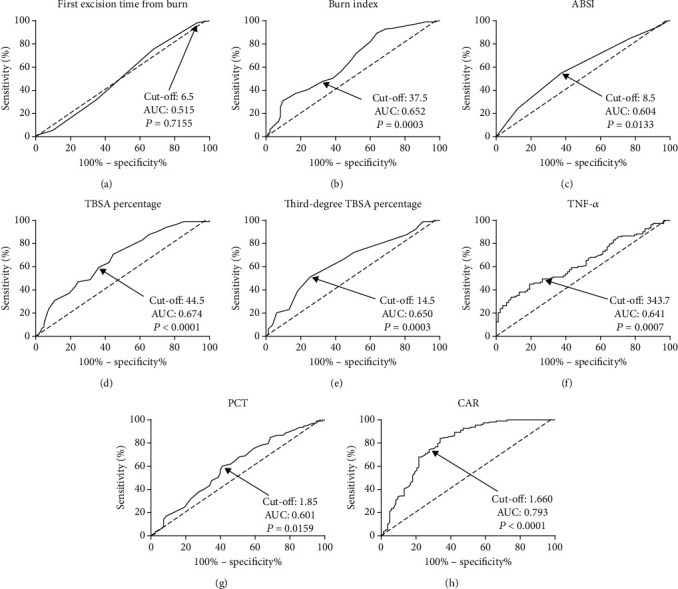
Predictive values of variables for sepsis in severe burns by ROC curve analysis. (a) First excision time from burn; (b) burn index; (c) ABSI; (d) TBSA percentage; (e) third-degree TBSA percentage; (f) TNF-*α*; (g) PCT; (h) CAR. CAR at admission was the most significant predictor with a cut-off value of 1.66, an AUC of 0.793, a sensitivity of 74.34%, and a specificity of 72.29% (*P* < 0.0001). CAR: C-reactive protein-to-albumin ratio; ABSI: abbreviated burn severity index; TBSA: total burn surface area; PCT: procalcitonin; TNF-*α*: tumor necrosis factor-*α*; ROC: receiver operating characteristic; AUC: area under the curve.

Of the potential risk factors (*P* < 0.05 in Tables 2 and 3), the predictive power of continuous variables for sepsis was evaluated by ROC curves and the results are displayed in Figure 2. The first excision time from burn was not a significant predictor (*P* = 0.716, Figure 2(a)), while burn index (*P* = 0.0003, Figure 2(b)), ABSI (*P* = 0.013, Figure 2(c)), TBSA percentage (*P* < 0.0001, Figure 2(d)), third-degree TBSA percentage (*P* = 0.0003, Figure 2(e)), TNF-*α* (*P* = 0.0007, Figure 2(f)), and PCT (*P* = 0.016, Figure 2(g)) were all predictors for sepsis in severe burns. Among these factors, the CAR at admission was the most significant predictor with a cut-off value of 1.66, an area under the curve (AUC) of 0.793, a sensitivity of 74.34%, and a specificity of 72.29% (*P* < 0.0001, Figure 2(h)).

### 3.3. Risk Factors for Sepsis and Outcomes

**Figure 3:**
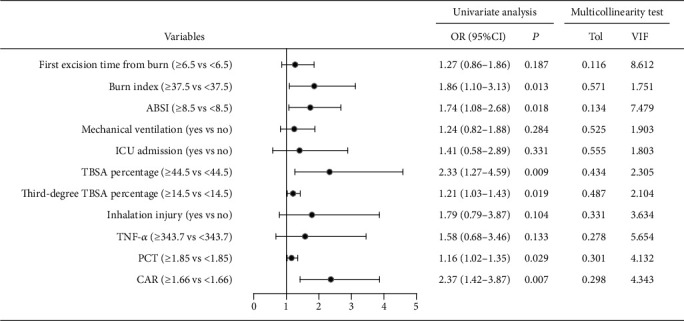
Forest plot of risk factors for sepsis by the univariate logistic regression analysis and multicollinearity test. ABSI: abbreviated burn severity index; TBSA: total burn surface area; ICU: intensive care unit; PCT: procalcitonin; TNF-*α*: tumor necrosis factor-*α*; CAR: C-reactive protein-to-albumin ratio; OR: odds ratio; CI: confidence interval; Tol: tolerance; VIF: variance inflation factor.

**Figure 4:**
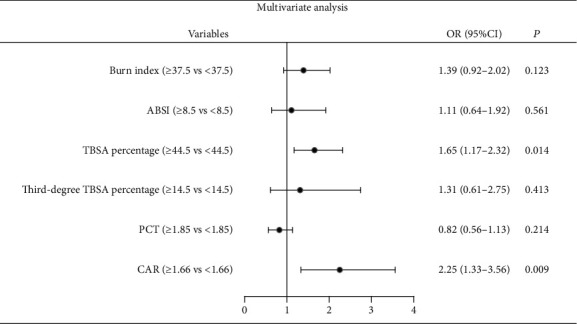
Forest plot of risk factors for sepsis by the multivariate logistic regression analysis. ABSI: abbreviated burn severity index; TBSA: total burn surface area; PCT: procalcitonin; CAR: C-reactive protein-to-albumin ratio; OR: odds ratio; CI: confidence interval.

The potential risk factors for sepsis in severe burns were investigated by the univariate and multivariate logistic regression analyses. No significant linearity was observed between sepsis and factors by multicollinearity test including VIF (Figure 3). As shown in Figure 3, six variables were potential risk factors by the univariate logistic regression analysis and were then placed into the multivariate model. TBSA percentage (OR: 1.65, 95% CI: 1.17-2.32, *P* = 0.014) and CAR at admission (OR: 2.25, 95% CI: 1.33-3.56, *P* = 0.009) were the two independent risk factors for sepsis in severe burns (see Figure 4). Of the 83 severe burns with sepsis, 26 died with a mortality rate of 31.3%. The causes of mortality were septic shock (*n* = 11), acute respiratory distress syndrome (ARDS, *n* = 6), intractable heart failure (*n* = 5), and others (*n* = 4).

### 3.4. CAR at Admission and Survival

**Figure 5:**
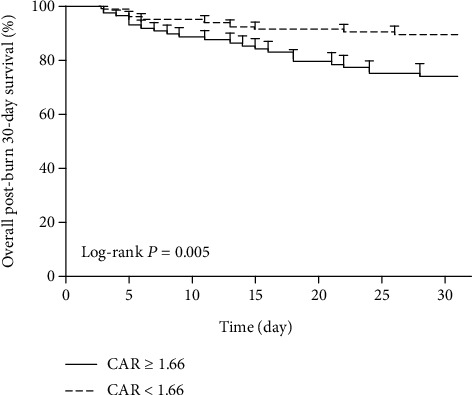
CAR at admission associated with postburn 30-day mortality by the Kaplan–Meier survival analysis. CAR: C-reactive protein-to-albumin ratio.

A Kaplan–Meier survival plot was generated to evaluate the association between CAR at admission and postburn 30-day survival. As indicated in Figure 5, a higher CAR level (≥1.66) at admission suggests a lower postburn 30-day survival rate (log-rank *P* = 0.005).

## 4. Discussion

Currently, sepsis is newly defined as a type of life-threatening organ dysfunction, which results from a dysregulated host response to infection [20]. As described by previous reports, sepsis is a complicated immune response, with the characteristics of massive inflammatory mediators in the early stage followed by the rapidly developed immunocyte impairment and immunosuppression state [21]. Significantly elevated inflammatory cytokines due to the excessive inflammation in early sepsis often result in the development of organ injury and dysfunction, ultimately causing death [22]. Thus, early prediction of sepsis and relevant intervention are critically important for improving outcomes in severe burns. Age, inhalation injury, and burned percent of TBSA are widely reported as the cornerstones for predictive scoring models [23, 24] in patients with burns. Our results also indicated TBSA as an independent risk factor for sepsis in severe burns. A recent study by Sheckter et al. [25] identifies that the percentage of TBSA was significantly associated with adverse outcomes in burn patients, which is quite in accordance with our results.

To our knowledge, this study firstly highlighted CAR at admission as an independent risk factor for sepsis and a novel prognostic factor among patients with severe burn injuries. Numerous studies have demonstrated a close correlation between CRP and sepsis. CRP is proposed to serve as a screening biomarker for neonatal sepsis [26]. A review in elderly geriatric patients by Ticinesi et al. [27] indicates that CRP expression at admission is helpful for acute infection detection, particularly sepsis. Another study by Liu et al. [28] suggests that the combination of PCT and CRP is helpful for the early diagnosis of pneumonia and sepsis, as well as treatment response measurement and prognosis prediction in neonates. A meta-analysis by Tan et al. [29] also indicates the diagnostic role of CRP for sepsis in adult patients, although with lower accuracy and specificity when comparing with PCT. Besides, the correlation between Alb and sepsis has also attracted a lot of attention. Reduced serum Alb concentrations are widely observed in patients with inflammatory states, including sepsis, due to the distribution alternation of Alb between extravascular and intravascular compartments induced by increased microvascular permeability [30]. Importantly, hypoalbuminemia correlates closely with increased mortality rates during hospitalization in intensive care units [31]. Besides the action as a plasma volume expander, Alb attracts more attention as the mediators of proinflammatory molecules and inflammation deserve more attention [32]. Alb is an important element in the moderation of the inflammatory response to bacterial infections by pathogen-associated molecular pattern- (PAMP-) albumin complexes via binding peptidoglycan, lipoteichoic acid, and lipopolysaccharide [33, 34]. Moreover, increased extravascular fluids induced by hypoalbuminemia also lead to some complications, such as healing abnormalities, edema, and increased susceptibility to sepsis [35]. In addition, excessive oxidative damage extensively exists in sepsis and often leads to cell dysfunction, death, and organ failure [36], while Alb acts as the main defense against this oxidative stress [37]. We hypothesized that CAR, which combines CRP and Alb, would imply the systemic inflammatory, nutritional, and immune statuses of patients. CAR could be much more sensitive, effective, and consistent than CRP or Alb alone. With the advantages, CAR has been widely recognized as a prognostic factor in various diseases, including metastatic renal cell carcinoma [38], ST elevation myocardial infarction [39], and hepatocellular carcinoma after curative resection [40]. CAR is reported to be an independent prognostic factor for 90-day mortality in septic individuals [41], which is in support of our conclusions. The close associations between CRP, Alb, and sepsis discussed above might be possible explanations for the predictive role of CAR for sepsis.

## 5. Conclusions

In conclusion, our results demonstrated that CAR at admission and TBSA percentage independently predicted sepsis in severe burns. A higher CAR (≥1.66) at admission was associated with an increased postburn 30-day mortality.

## Figures and Tables

**Table 1:** Clinical and therapeutic parameters in severe burns admitted to the intensive care unit (ICU).

Parameters	Severe burns admitted to ICU (*n* = 135)
Shock, *n* (%)	74 (54.8)
Sepsis, *n* (%)	81 (60.0)
Septic shock, *n* (%)	29 (21.5)
Vasoactive drugs, *n* (%)	90 (66.7)
Blood transfusion	—
Red blood cell (u)	7.4 ± 1.6
Plasma (ml)	4740 ± 720
Platelet (therapeutic amount)	1.50 ± 0.25
Cryoprecipitate (u)	0.14 ± 0.02

**Table 2:** Clinical parameters associated with sepsis in severe burns.

Parameters	Sepsis		*P* value
	Yes (*n* = 83)	No (*n* = 113)	
Age (years)	41.8 ± 8.1	43.1 ± 7.5	0.248
Gender, *n* (%)	—	—	0.427
Male	49 (59.0)	73 (64.6)	—
Female	34 (41.0)	40 (35.4)	—
BMI (kg/m^2^)	22.3 ± 2.2	22.2 ± 2.5	0.771
Causes	—	—	0.908
Thermal	68 (81.9)	90 (79.6)	—
Chemical	10 (12.0)	16 (14.2)	—
Electronic	5 (6.0)	7 (6.2)	—
Inhalation injury, *n* (%)	30 (36.1)	24 (21.2)	0.021^∗^
Admission time from burn (h)	18.2 ± 3.9	17.9 ± 4.2	0.611
Number of operations, *n* (%)	4.3 ± 0.4	4.2 ± 0.5	0.125
Mean duration of operation (min)	93.4 ± 17.8	90.3 ± 20.1	0.251
First excision time from burn (d)	5.0 ± 1.1	4.7 ± 0.9	0.037^∗^
Burn index	41.4 ± 6.3	38.8 ± 5.8	0.003^∗^
ABSI	8.8 ± 1.1	8.3 ± 1.0	0.001^∗^
APACHE II score at admission	11.0 ± 1.6	10.7 ± 1.2	0.135
SOFA score at admission	3.2 ± 0.7	3.1 ± 0.6	0.284
Mechanical ventilation, *n* (%)	38 (45.8)	30 (26.5)	0.005^∗^
ICU admission, *n* (%)	81 (97.6)	54 (47.8)	<0.001^∗^
Preoperative comorbidities, *n* (%)	—	—	—
Diabetes mellitus	9 (10.8)	11 (9.7)	0.800
Hypertension	14 (16.9)	17 (15.0)	0.730
CCD	6 (7.2)	9 (8.0)	0.848
COPD	5 (6.0)	7 (6.2)	0.961
TBSA percentage, *n* (%)	47.3 ± 8.1	42.5 ± 7.4	<0.001^∗^
Third-degree TBSA percentage, *n* (%)	15.9 ± 3.7	14.6 ± 2.9	0.006^∗^

BMI: body mass index; ABSI: abbreviated burn severity index; APACHE II: Acute Physiology and Chronic Health Evaluation II; SOFA: Sequential Organ Failure Assessment; ICU: intensive care unit; CCD: chronic coronary disease; COPD: chronic obstructive pulmonary disease; TBSA: total burn surface area. *P* values were calculated by Student's *t*-test or chi-squared test. ^∗^*P* < 0.05.

**Table 3:** Laboratory tests associated with sepsis in severe burns.

Laboratory tests at admission	Sepsis		*P* value
	Yes (*n* = 83)	No (*n* = 113)	
Hb (g/L)	116.9 ± 9.6	118.9 ± 8.8	0.132
Plt (×10^9^/L)	282.1 (82.0, 662.6)	269.8 (88.0, 722.3)	0.543
WBC (×10^9^/L)	10.1 ± 2.3	9.7 ± 1.9	0.185
Hct	0.41 ± 0.09	0.42 ± 0.08	0.413
TNF-*α* (pg/mL)	358.4 ± 32.3	344.8 ± 40.2	0.012^∗^
PCT (*μ*g/L)	2.3 (0.05, 130.3)	1.2 (0.05, 114.0)	<0.001^∗^
Creatinine (*μ*mol/L)	88.0 (66.0, 134.0)	79.0 (62.0, 112.0)	0.324
BUN (mmol/L)	7.7 (4.5, 9.3)	6.9 (4.8, 8.9)	0.278
CRP	55.3 ± 20.1	47.8 ± 16.5	0.005^∗^
Alb	34.6 ± 3.6	35.9 ± 4.5	0.031
CAR	2.14 (0.03, 3.84)	1.27 (0.01, 2.72)	<0.001^∗^

Hb: hemoglobin; Plt: platelet; WBC: white blood cell; Hct: hematocrit; PCT: procalcitonin; BUN: blood urea nitrogen; TNF-*α*: tumor necrosis factor-*α*; CAR: C-reactive protein-to-albumin ratio. *P* values were calculated by Student's *t*-test or Mann–Whitney *U* test. ^∗^*P* < 0.05.

## Data Availability

Please contact the corresponding author (Jiliang Li, email: lijiliang_nb@yeah.net).
